# Triglyceride/HDL Ratio as a Screening Tool for Predicting Success at Reducing Anti-Diabetic Medications Following Weight Loss

**DOI:** 10.1371/journal.pone.0069285

**Published:** 2013-07-15

**Authors:** Ghanshyam Palamaner Subash Shantha, Anita Ashok Kumar, Scott Kahan, Pavan Kumar Irukulla, Lawrence Jay Cheskin

**Affiliations:** 1 Department of Epidemiology, Johns Hopkins Bloomberg School of Public Health, Baltimore, Maryland, United States of America; 2 Department of Internal Medicine, The Wright Center for Graduate Medical Education, Scranton, Pennsylvania, United States of America; 3 Department of Health Policy, Johns Hopkins Bloomberg School of Public Health, Baltimore, Maryland, United States of America; 4 Department of Internal Medicine, The Wright Center for Graduate Medical Education, Scranton, Pennsylvania, United States of America; 5 Department of Health, Behavior and Society, Johns Hopkins Bloomberg School of Public Health, Baltimore, Maryland, United States of America; 6 Department of Health Policy, George Washington University School of Public Health and Health Services, Washington, D.C., United States of America; 7 Johns Hopkins Weight Management Center, Johns Hopkins University, Baltimore, Maryland, United States of America; Glaxo Smith Kline, Denmark

## Abstract

**Background and Objectives:**

Intentional weight loss, by reducing insulin resistance, results in both better glycemic control and decreased need for anti-diabetic medications. However, not everyone who is successful with weight loss is able to reduce anti-diabetic medication use. In this retrospective cohort study, we assessed the predictive accuracy of baseline triglyceride (TGL)/HDL ratio, a marker of insulin resistance, to screen patients for success in reducing anti-diabetic medication use with weight loss.

**Methods:**

Case records of 121 overweight and obese attendees at two outpatient weight management centers were analyzed. The weight loss intervention consisted of a calorie-restricted diet (~1000Kcal/day deficit), a behavior modification plan, and a plan for increasing physical activity.

**Results:**

Mean period of follow-up was 12.5 ± 3.5 months. By study exit, mean weight loss and mean HbA1c% reduction were 15.4 ± 5.5 kgs and 0.5 ± 0.2% respectively. 81 (67%) in the study cohort achieved at least 1 dose reduction of any anti-diabetic medication. Tests for predictive accuracy of baseline TGL/HDL ratio ≤ 3 to determine success with dose reductions of anti-diabetic medications showed a sensitivity, specificity, positive predictive value, negative predictive value, area under the curve, likelihood ratio (LR) + and LR-of 81, 83, 90, 70, 78, 4.8 and 0.2, respectively. Reproducibility of TGL/HDL ratio was acceptable.

**Conclusion:**

TGL/HDL ratio shows promise as an effective screening tool to determine success with dose reductions of anti-diabetic medications. The results of our study may inform the conduct of a systematic review using data from prior weight loss trials.

## Introduction

In the last decade, the prevalence of obesity [body mass index (BMI; calculated as weight in kilograms divided by height in meters-squared) ≥ 30], among adult Americans was 35%, and is expected to increase in years to come [1]. Type 2 diabetes mellitus (DM) is an important problem among the obese. Overweight (BMI 25–29.9), obese (BMI 30–39.9), and extremely obese (BMI > 40) American adults had 1.59, 3.44 and 7.37 times higher odds, respectively, of diagnosed DM [2]. Obesity and DM in unison create enormous health care costs, of which the direct and indirect costs of pharmacotherapy plays a major role [3].

Intentional weight loss, by decreasing insulin resistance, is associated with better glycemic control [4,5] and consequently a decrease in the need for anti-diabetic medications [6]. However, it is well known that not everyone who loses weight is successful in reducing anti-diabetic medications [6]. Hence, a clinically applicable screening tool that can predict success with dose reductions of anti-diabetic medications would be useful, and could help motivate patients, as well as assist practitioners in patient counseling, and individualizing the intensity of weight loss intervention for a given obese patient attempting weight loss. Since insulin resistance is the central pathophysiology behind obesity-associated diabetes, we hypothesized that a marker of insulin resistance, measured at the onset of weight loss efforts, might serve as a screening tool to predict success with dose reductions of anti-diabetic medications accompanying successful weight loss. Tryglyceride (TGL)/HDL ratio is a known marker of insulin resistance [7]. To test this hypothesis, we studied overweight and obese patients from 2 University-based weight management programs, analyzing the relationship between triglyceride/HDL ratio and success in reducing anti-diabetic medications.

## Materials and Methods

### Study setting and design

This retrospective cohort study was conducted in two, University based, specialty, outpatient weight management clinics, the Johns Hopkins Weight Management Center in Baltimore, MD, and the George Washington Weight Management program in Washington, DC. Institutional review board approval was obtained from Johns Hopkins University institutional review board for conducting the study. Informed consent was not obtained from the participants, as the data was analyzed anonymously per institutional review board guidelines. Institutional review board waived the need for written informed consent from the participants.

Case records of patients with BMI > 25 kg/m^2^ at the time of enrollment into the two weight management programs during the period March 2008 to January 2012 were assessed for eligibility. The study cohort consisted of patients with a diagnosis of DM at the time of enrollment who reported taking anti-diabetic medications. Patients were excluded if they did not have a diagnosis of DM at the time of enrollment into the programs.

### Baseline data collection

Demographic data (age, gender, race/ethnicity), cardiovascular risk factors (smoking, diabetes, hypertension), medication history (anti-diabetic medications, anti-hypertensive medications, and lipid-lowering drugs), clinical parameters (height, weight, systolic blood pressure (SBP) and diastolic blood pressure (DBP), and laboratory parameters (fasting glucose, HbA1c, total cholesterol, low-density lipoprotein cholesterol (LDL), high-density lipoprotein cholesterol (HDL), triglycerides) were collected from the study cohort. BMI was calculated as per standard guidelines [8]. DM was identified by physician diagnosis.

### Patient follow-up and weight management intervention details

The weight loss intervention protocols followed at the two participating clinics were similar, and consisted of team-based, comprehensive evaluation and treatment for weight loss. The study participants had physician visits for follow-up twice a month on average. The baseline visit consisted of a physician-conducted medical history and physical examination, blood tests (as described above), and detailed dietary, behavioral, and exercise evaluations. Treatment was individualized, but typically consisted of an approximately 1000 kcal/day energy-deficit diet, often utilizing meal replacements, a behavior modification plan, and a plan for increasing physical activity utilizing both aerobic exercise and strength training. These interventions with diet, physical activity and behavior modification were similar in both participating institutions. Depending on treatment response, the intervention was tailored to address individual patient needs. The decision to alter the dose of or discontinue anti-diabetic medications was based on the clinical judgment of the treating physicians. Factors considered in deciding on dose reductions included magnitude of weight loss, glycemic control, hypoglycemic symptoms, and the patient’s compliance with the weight management protocol.

### Triglyceride/HDL ratio cutoffs tested as screening tool

Triglyceride/HDL ratios of all study cohort participants at the time of their enrollment into the weight management programs were calculated. Cutoffs for triglyceride/HDL ratio of ≤ 2, ≤ 2.5, ≤ 3, ≤ 3.5, and ≤ 4.0 were evaluated to determine which cut-off value yielded optimal results as a screening tool. Using these baseline triglyceride/HDL cutoffs, each member of the study cohort was categorized, within each cutoff category, as successful or unsuccessful in achieving at least one dose reduction of any anti-diabetic medication by study exit. Patients exited the study if they achieved at least 1 dose reduction of any of their anti-diabetic medications, or at study conclusion in January 2012. One dose reduction was defined as any recorded decrease in dose of anti-diabetic medication, which included lowering the dose or completely stopping that particular medication.

### Testing reproducibility of the triglyceride/HDL ratio

Three of the authors were involved in this experiment. One of them was an internist with 5 years’ post medical school experience; one was a MD with 3 years’ post medical school experience and third was a diabetes educator with 1 year post bachelors degree experience. All three authors received a brief 20 minutes training regarding calculation of triglyceride/HDL ratio. A computer generated random sample (n = 60) from the study cohort was selected. First the internist calculated baseline triglyceride/HDL ratio for this random study cohort patients, blinded with regard to patient and medication history. To test intra-observer correlation, the internist recalculated baseline triglyceride/HDL ratio, for the same random sample of study cohort participants after 1 week, blinded with regard to earlier triglyceride/HDL ratio results, patient and medication history. Correlation was calculated comparing the present and previous findings of the internist. Then, the MD calculated baseline triglyceride/HDL ratio for the same random sample used by the internist, blinded with regard to patient, medication details and triglyceride/HDL ratios. Correlation was calculated comparing the triglyceride/HDL ratios of the internist with that of the MD. Similarly, the diabetes educator calculated triglyceride/HDL ratio for the same random sample and correlation was calculated comparing her findings with that of the internist.

### Statistical analysis

Data was expressed in number (%) for categorical variables, and as mean ± standard deviation for continuous variables. The baseline characteristics of study cohort participants who successfully dose reduced anti-diabetic medications were compared with the study cohort participants who were not successful with even 1 dose reduction of any anti-diabetic medications, using Student’s t test and chi-squared tests, as appropriate. Similarly, these 2 groups were compared with regard to magnitude of weight loss and HbA1C% reduction by study exit.

Multiple linear regression analysis and multiple logistic regression analysis were performed with magnitude of weight loss (kg) and dose reductions of anti-diabetic medications (coded as a dichotomous variable) as dependent variables. Age, gender, hypertension diagnosis, smoking, lipid lowering medication use, duration of diabetes, baseline BMI and TGL/HDL ratio were independent variables. In addition, logistic regression analysis assessing association between dose reductions of anti-diabetic medications and TGL/HDL ratio was adjusted for magnitude of weight loss.

To assess if triglyceride/HDL ratio is an effective screening tool to predict success with dose reductions of anti-diabetic medications, sensitivity, specificity, positive predictive value, negative predictive value, likelihood ratio of positive test, likelihood ratio of negative test and area under the receiver operating characteristics curve (ROC) were calculated, using standard methods, for the cut-off points for triglyceride/HDL ratios [9]. Also, TGL/HDL ratio was coded as continuous variable and AUC was computed. Kappa statistics was used to assess intra- and inter-observer agreement in interpreting triglyceride/HDL ratios [9]. A P-value of <0.05 was considered statistically significant. Statistical analyses were performed using Stata 11.0 statistical software [10].

## Results

In total, 179 patient records (107 from Johns Hopkins and 72 from George Washington) were identified and reviewed. Of these, 58 (32%) were excluded (excluded cohort) because they did not have a diagnosis of diabetes at the time of enrollment into the programs. The remaining 121 (68%) with a diagnosis of diabetes formed the study cohort. All study cohort patients were taking one or more anti-diabetic medications at the time of enrollment. Demographic characteristics and medication details of the study cohort and excluded cohort are shown in Table 1. Follow-up details of the study cohort by study exit are detailed in Table 2.

**Table 1:** Baseline characteristics of the study cohort.

Variables	Study cohort	Successful#	Not successful*	P-value
	(n = 121)	(n = 81)	(n = 40)	
Age (yrs)	51.8 ± 8.9	52.7 ± 9.1	53.7 ± 9.3	0.133
Males –n (%)	67 (55)	45 (56)	22 (55)	0.668
Caucasians –n (%)	90 (74)	63 (78)	27 (68)	0.042
African Americans –n (%)	31 (26)	18 (22)	13 (32)	0.044
Current smoking –n (%)	24 (20)	16 (20)	8 (20)	0.996
Duration of diabetes (yrs)	7.5 ± 2.5	8.0 ± 2.0	7,5 ± 2.0	0.371
Mean baseline weight (Kgs)	126.5 ± 18.2	127.1 ± 16.4	126.9 ± 19.3	0.417
Mean BMI (kg/m^2^)	36.1 ± 6.2	36.1 ± 5.5	35.6 ± 4.7	0.226
BMI: 25-30 –n (%)	30 (25)	23 (28)	7 (18)	0.047
BMI: 31-40 –n (%)	64 (53)	51 (63)	13 (33)	0.021
BMI ≥ 41 – n (%)	27 (22)	7 (9)	20 (50)	0.017
HbA1C %	8.2 ± 1.6	8.1 ± 2.3	8.0 ± 1.7	0.179
Mean TC (mmol/l)	5.2 ± 1.3	5.2 ± 1.1	5.1 ± 1.7	0.113
Mean LDL – C (mmol/l)	4.2 ± 1.3	4.0 ± 1.4	4.1 ± 1.2	0.091
Mean HDL – C (mmol/l)	1.2 ± 0.4	1.1 ± 0.3	1.1 ± 0.4	0.216
Mean TGL (mmol/l)	1.7 ± 0.3	2.0 ± 0.5	1.9 ± 0.6	0.166
Mean TGL/HDL ratio	2.9 ± 1.2	3.1 ± 0.8	2.8 ± 1.1	0.051
HTN diagnosis –n (%)	102 (84)	69 (85)	33 (83)	0.067
MS –n (%)	68 (56)	45 (56)	23 (58)	0.116
Lipid lowering drugs –n (%)	91 (75)	64 (79)	27 (68)	0.055
Statins	91 (75)	64 (79)	27 (68)	0.055
Atorvastatin	52 (43)	27 (33)	25 (63)	0.172
Pravastatin	24 (20)	13 (16)	11 (28)	0.133
Rosuvastatin	15 (12)	8 (10)	7 (18)	0.226
Fenofibrate	7 (6)	4 (5)	3 (8)	0.417
Anti – HTN drugs –n (%)	102 (84)	69 (85)	33 (83)	0.067
Metformin-n (dose/day)	71 (1.7g)	51 (1.7g)	20 (1.7g)	0.044
Sulphonylureas-n (%)	59 (49)	40 (49)	19 (48)	0.133
Glyburide-n (dose/day)	24 (10mg)	16 (10mg)	8 (10mg)	0.091
Glipiside-n (dose/day)	21 (10mg)	12 (10mg)	9 (10mg)	0.046
Glymipride-n (dose/day)	14 (2-4 mg)	9 (2-4 mg)	5 (2-4 mg)	0.117
Sitagliptin-n (dose/day)	19 (100 mg)	11 (100 mg)	8 (100 mg)	0.061
Insulin-n (dose/day)	59 (60 U)	45 (65 U)	14 (60 U)	0.155

#Study cohort participants successful with dose reductions

* Study cohort participants not successful with dose reductions

**Table 2:** Follow-up details of the study cohort.

	Study cohort	Successful#	Not successful*	P value
Follow-up (months)	12.5 ± 3.5	13.0 ± 2.5	12.5 ± 2.5	0.511
Baseline weight (Kgs)	126.5 ± 18.2	127.1 ± 16.4	126.9 ± 19.3	0.417
Final weight (Kgs)	110.3 ± 5.9	108 ± 11.2	117 ± 5.7	0.031
Weight loss (Kgs)	15.4 ± 5.5	16.9 ± 4.7	9.2 ± 3.1	0.029
Baseline HbA1C%	8.2 ± 1.6	8.1 ± 2.3	8.0 ± 1.7	0.179
Final HbA1C%	7.7 ± 0.3	7.4 ± 0.6	7.9 ± 0.2	0.039
Change in HbA1C%	0.5 ± 0.2	0.7 ± 0.2	0.2 ± 0.1	0.035

#Study cohort participants successful with dose reductions

* Study cohort participants not successful with dose reductions

By study exit, 81 (67%) in the study cohort achieved at least 1 dose reduction of any anti-diabetic medication. The remaining 40 (33%) in the study cohort failed to achieve even 1 dose reduction of their anti-diabetic medication; their weight loss and HbA1c% reduction was significantly less than those in the study cohort who did successfully dose-reduce anti-diabetic medications (Table 2). 

Baseline BMI and TGL/HDL ratio had a strong and independent association with magnitude of weight loss and dose reductions of anti-diabetic medications (Table 3).

**Table 3:** Factors associated with magnitude of weight loss and success with dose reductions.

Variables	Magnitude of weight loss (Kgs)#		Dose reductions*	
	ß (95% C.I)	P value	OR (95% C.I)	P value
Age	1.57 (-0.11 – 3.11)	0.136	1.01 (0.77 – 2.32)	0.216
Gender	0.91 (-0.62 – 2.17)	0.133	1.15 (0.67 – 2.37)	0.210
Hypertension	2.45 (-0.12 – 3.16)	0.133	0.96 (0.42 – 1.92)	0.132
Smoking	1.45 (-1.50 – 3.63)	0.511	1.07 (0.66 – 2.11)	0.286
Lipid lowering medication use	0.97 (-1.10 – 2.36)	0.162	1.13 (0.71 – 1.96)	0.149
Baseline BMI	1.53 (0.58 – 2.27)	0.033	1.47 (1.14 – 2.35)	0.031
TGL/HDL ratio	5.28 (1.51 – 8.13)	0.014	1.31 (1.03 – 2.41)	0.022
Duration of diabetes (yrs)	1.10 (-2.74 – 3.71)	0.159	1.14 (0.68 – 2.66)	0.291
Magnitude of weight loss (kgs)	-	-	1.18 (1.09 – 1.96)	0.031

OR: Odds Ratio; β: Linear regression coefficient; C.I: confidence interval,

#multi-variate linear regression analysis

* multi-variate logistic regression analysis

Tests for TGL/HDL ratios as a screening tool to predict success with dose reductions of anti-diabetic medications is shown in Table 4. When TGL/HDL ratio was coded as a continuous variable AUC was 82. Of the selected cut-off values, TGL/HDL ratio ≤ 3 showed optimal results with a sensitivity, specificity, positive predictive value, negative predictive value, AUC, LR + and LR-of 81, 83, 90, 70, 78, 4.8 and 0.2 respectively (Table 4). ROC curves can be viewed in Figure S1 and Figure S2. Further, kappa statistics for intra-observer agreement was 0.99 and Inter-observer agreement between the internist and the MD was 0.99 and between the internist and the diabetes educator was 0.99.

**Table 4:** TGL/HDL ratio as a screening tool to predict success with dose reductions.

Parameters	TGL/HDL ratio				
	≤ 2.0	≤ 2.5	≤ 3.0	≤ 3.5	≤ 4.0
Sensitivity (%)	20	41	81	85	88
Specificity (%)	95	85	83	58	35
PPV	89	85	90	80	73
NPV	37	41	70	66	58
AUC	51	62	78	65	53
LR+	4.0	2.7	4.8	2.0	1.4
LR-	0.8	0.7	0.2	0.2	0.3
Number successful	16	33	66	69	71
Total	18	39	73	86	97

## Discussion

Our study of overweight and obese adults has identified TGL/HDL ratio as a potentially effective screening tool to predict success with dose reductions of anti-diabetic medications in patients who successfully lose weight. 

We found, not surprisingly, that magnitude of weight loss was an important factor determining success with dose reductions of anti-diabetic medications (Table 2). Mechanistically, weight loss is accompanied by decreasing insulin resistance and hence better glycemic control [11]. This could explain the strong and independent association of TGL/HDL ratio, a marker of insulin resistance [7], with magnitude of weight loss, and thus with the higher probability of dose reductions of anti-diabetic medications that was observed (Table 3). This reasoning comports with our rationale for the use of TGL/HDL ratio as a screening tool to predict success with dose reductions of anti-diabetic medications. Prior studies have also shown TGL/HDL ratio to positively correlate with adiposity, and its decrease proportional to magnitude of weight loss [12,13].

Further, 75% of our patients were using lipid lowering medications at baseline (Table 1). However, the observed independent association of TGL/HDL ratio with dose reductions of anti-diabetic medications even after adjusting for lipid lowering medication use (Table 3) highlights the potential use of TGL/HDL ratio as a screening tool to predict success with dose reductions of anti-diabetic medications irrespective of lipid lowering medication use.

We note that when a screening tool is assessed for its predictive accuracy, the prevalence of the event of interest should be taken into consideration [14]. When the prevalence of the event of interest (dose reductions of anti-diabetic medications) is high (67% in our study), values of sensitivity, specificity, positive predictive value and negative predictive value may be less useful for clinical application, since all of the above-mentioned indicators of test accuracy vary with prevalence. In this context, the indicators of test accuracy which do not significantly vary with prevalence are the most clinically relevant. LR+ and LR-do not vary with prevalence of the disease [14]. An LR+ >1 and an LR− <1 makes a diagnostic test clinically meaningful, while a LR+ >10 and LR− <0.1 indicates very high accuracy [14]. In our study, among all arbitrary TGL/HDL ratio cut-offs assessed, TGL/HDL ratio ≤ 3, with its LR+ of 4.8 and LR-was 0.2, had the highest predictive accuracy. Area under the ROC curve of 78% also conveys the same meaning. Combined with its good reproducibility, TGL/HDL ratio may well be a clinically useful screening tool for this purpose. Interestingly, TGL/HDL ratio has been found in other studies to be an effective tool for predicting gestational diabetes [15], cardiovascular risk in patients with familial hypercholestrolemia [16], atherogenic apolipoprotein B levels [17] and as a surrogate marker in the mechanistic pathway through which pioglitazone possibly delays coronary atheroma progression in diabetic patients [18].

### Limitations

We observed significantly greater magnitudes of weight loss (15.4 kgs by 15 months of follow-up) compared to typical randomized, controlled weight loss trials (RCTs) (weight loss: 4–6 kgs within 12-15 months of follow-up, SBP/DBP: 3-4/2-3 mmHg) [19,20]. It is thus possible that confounding due to unknown factors may have played a role. Or, surveillance bias is possible, due to the intensity of weight loss intervention being individualized, and the decision to dose reduce being subjective and at the discretion of the treating physicians. Also, small sample size limited us from analyzing our research question with individual anti-diabetic medications and individual BMI classes. Further, convenience sampling may have led to selection bias. Retrospective cohort design limited us to what was recorded in patient charts; for example, we did not have date on waist/hip ratio, which might also have relevance as a predictor.

## Conclusions

In conclusion, TGL/HDL ratio was found to show promise as a clinically applicable screening tool. It showed moderately high accuracy in predicting success in reducing use of anti-diabetic medications among overweight and obese diabetics attempting weight loss. Further, it is easy to calculate and has good reproducibility. Before advocating widespread use, this tool should be prospectively validated in future RCTs or clinical settings, and evaluated in systematic reviews using data that is available from completed RCT’s [19,20].

## Supporting Information

Figure S1 ROC curve for TGL/HDL ratio ≤ 3 as a screening tool to predict success with dose reductions of anti-diabetic medications. (DOC)

Figure S2 ROC curve when TGL/HDL ratio was coded as continuous variable. (DOC)
