# Supplementary material for: Triglyceride/HDL Ratio as a Screening Tool for Predicting Success at Reducing Anti-Diabetic Medications Following Weight Loss
Source: PLoS One. 2013 Jul 15;8(7):e69285. doi: 10.1371/journal.pone.0069285 (PMC3712020; doi:10.1371/journal.pone.0069285)
Supplement: Figure S1 — (DOC) [file pone.0069285.s001.doc]

**Figure S1: ROC curve for TGL/HDL ratio ≤ 3 as a screening tool to predict success with dose reductions of anti-diabetic medications**
